# P-1417. Correlation Between Imipenem Susceptibility and Clinical Response in Patients with Extra-Pulmonary Rapidly Growing Non-Tuberculous Mycobacterium Infections

**DOI:** 10.1093/ofid/ofaf695.1604

**Published:** 2026-01-11

**Authors:** Wantin Sribenjalux, Waewta Kuwatjanakul, Atibordee Meesing, Natapong Manomaiwong, Chanikarn Sinkijcharoenchai, Yotsakorn Leelapaiboon, Passapong Susangrat, Rujipas Chuenchomkit, Kornkamon Thamwiwat, Aunshistha Suebwattanapongkul

**Affiliations:** Faculty of Medicine, Khon Kaen University, Khon Kaen, Khon Kaen, Thailand; Faculty of Medicine, Khon Kaen University, Khon Kaen, Khon Kaen, Thailand; Faculty of Medicine, Khon Kaen University, Khon Kaen, Khon Kaen, Thailand; Faculty of Medicine, Khon Kaen University, Khon Kaen, Khon Kaen, Thailand; Faculty of Medicine, Khon Kaen University, Khon Kaen, Khon Kaen, Thailand; Faculty of Medicine, Khon Kaen University, Khon Kaen, Khon Kaen, Thailand; Faculty of Medicine, Khon Kaen University, Khon Kaen, Khon Kaen, Thailand; Faculty of Medicine, Khon Kaen University, Khon Kaen, Khon Kaen, Thailand; Faculty of Medicine, Khon Kaen University, Khon Kaen, Khon Kaen, Thailand; Faculty of Medicine, Khon Kaen University, Khon Kaen, Khon Kaen, Thailand

## Abstract

**Background:**

Extrapulmonary Nontuberculous Mycobacterium (NTM) infections are an understudied condition with limited comprehensive data. Rapidly growing NTM (RGM) species account for a significant proportion of cases, and imipenem plays a crucial role in the induction phase of treatment. However, no studies have examined the relationship between imipenem susceptibility and clinical outcomes, nor the factors influencing prognosis after imipenem treatment.

**Figure:**
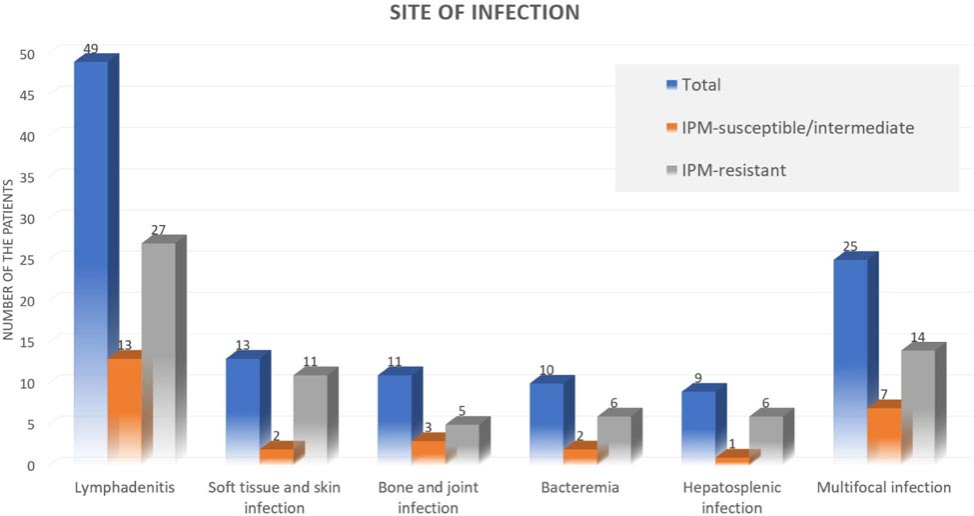

**Figure:**
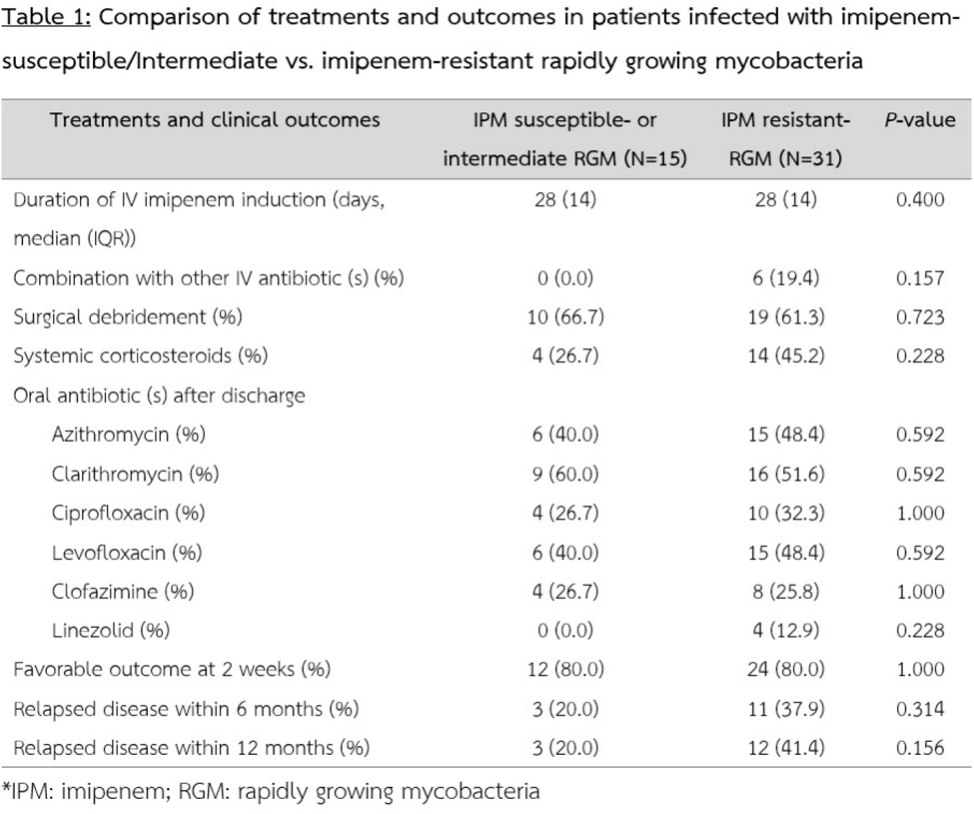

**Methods:**

This retrospective descriptive study analyzed secondary data from medical records at Srinagarind Hospital (Khon Kaen University, Thailand) between February 27, 2015, and December 15, 2022. We included patients who were diagnosed with extrapulmonary NTM infection based on ICD-10 codes and confirmed by culture for RGM.

**Figure:**
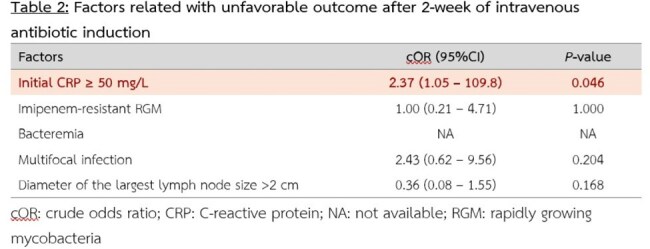

**Results:**

A total of 56 patients were included, with a majority being female (57.1%) and a mean age of 53.6 years. The most common clinical presentation was lymphadenitis (87.5%), and the most prevalent risk factor was the presence of interferon-gamma autoantibodies (78.2%). Among the RGM isolates tested, 15 (26.8%) were classified as susceptible or intermediate, 31 (55.4%) were resistant to imipenem, and the remaining isolates had no imipenem susceptibility results. There was no significant difference in the proportion of patients with favorable clinical outcomes at 2 weeks after treatment (80% vs. 80%, p = 1.000) or in recurrence rates at 12 months (20% vs. 41.6%, p = 0.156) between the susceptible/intermediate and resistant groups. However, an initial C-reactive protein (CRP) level ≥50 mg/L was significantly associated with unfavorable clinical outcomes following intravenous antibiotic treatment.

**Conclusion:**

Imipenem susceptibility in rapidly growing NTM species was not significantly associated with short-term clinical outcomes or disease recurrence within one year. However, an elevated initial CRP level was an important predictor of treatment response at 2 weeks.

**Disclosures:**

All Authors: No reported disclosures

